# Multiple co-frequency sources DOA estimation for coprime vector sensor arrays

**DOI:** 10.1371/journal.pone.0285459

**Published:** 2023-05-09

**Authors:** Xiao Chen, Hao Zhang, Zhen Wang, Yujie Chen, Yong Gao

**Affiliations:** 1 Department of Electronic Engineering, Ocean University of China, Qingdao, China; 2 Department of Electrical and Computer Engineering, University of Victoria, Victoria, BC, Canada; TU Wien: Technische Universitat Wien, AUSTRIA

## Abstract

For the problem of direction-of-arrival (DOA) estimation using a coprime array, there are high spatial spectrum outputs of false alarms caused by the overlap of main and grating lobes from subarrays. In this paper, a DOA estimation method of more than two co-frequency sources for a coprime vector hydrophone array is proposed. The method is based on vector cross terms (VCTs), making full use of the directivity of channel combinations for vector hydrophones. Based on VCTs, the characteristic data point identification method is conducted and ensures that the bearing data with the characteristic can be preserved. For further interference rejection, the paper designs Queue Selection (QS) method based on inverse beamforming. The influence of grating lobes can be weakened with the QS, further improving the accuracy of direction extraction. The algorithm in this work does not require decoherence processing, and the simulation work shows that it achieves stable DOA estimation with a low signal-to-noise ratio (SNR).

## Introduction

Coprime linear arrays (CLAs) have become popular nowadays for providing system array configurations beyond Nyquist sampling and reducing mutual coupling between array elements [1–17]. The classical spatial spectrum estimation algorithms for CLAs are based on Product [18] and Min [19] theories. The Product theory calculates the spatial power spectral density (PSD) of the observed signal through multiplication. The multiplication is conducted by a beamformed subarray scanned response with the complex conjugate of another. The Min processor estimates the spatial PSD by selecting the lowest value from the CLA subarray periodograms for each bearing. The Product method can resolve the spatial aliasing ambiguity arising from CLA subarrays, and the Min processor maintains identical array resolution while attaining the reduced height of peak sidelobe and total sidelobe area with the same extended CLA geometry.

Direction of arrival (DOA) estimation is a vital area of array signal processing in acoustics, radar, and wireless communications [20]. For the issue of DOA estimation of CLAs, the DOA estimation method obtained by combining the multiple signal classification (MUSIC) results of the linear arrays (DECOM) utilizes a combination of the MUSIC results from two decomposed uniform linear arrays to obtain DOA estimates [21]. An underwater vector sensor coprime line array (vCLA) with an extended configuration and sound pressure and vibration velocity joint processing (PVJP) was proposed for improving beamforming and DOA estimation performance [22]. The DECOM method and the extended vCLA are based on the physical structure of the array itself, as opposed to some methods, based on research conducted in the virtual array element domain. For example, a new DOA estimation algorithm utilizing Toeplitz matrix reconstruction and virtual array interpolation methods was introduced [23]. A discrete inverse Fourier transform (IDFT) method employed virtual signals of second order with angular-spatial frequencies to estimate the DOA information [24]. These methods focus on the advantage of high degrees of freedom (DOFs) using the virtual array elements and are primarily used in the radar field. In [25], a technique was presented that utilized a coprime electromagnetic vector-sensor (EMVS) array. The method relied on solving a nuclear norm minimization (NNM) problem to construct a larger covariance matrix that was then used for DOA estimation. Then the problem of DOA estimation in two dimensions was discussed for a bistatic multiple-input multiple-output (MIMO) radar system with coprime EMVS arrays in [26]. There is also some research about DOA estimation in different scenarios for virtual array domain processing. A multi-source DOA tracking method was presented [27]. The number of sources was estimated using the minimum description length (MDL), and then the MUSIC pseudo-spectrum was utilized. For impulsive noise and non-circular signals scenarios, the DOA estimation algorithm for a coprime array was shown [28], which proposed an augmented phased fractional low-order moment (A-PFLOM) to suppress noise and applied reduced-dimension MUSIC (RD-MUSIC) subspace techniques for DOA information. For mixed noise scenarios, a DOA estimation method based on phased fractional low-order moment was advanced [29]. For the scenario of impulsive noise, fractional low-order moments were applied [30].

Nevertheless, CLAs suffer from significant limitations in practical applications. High grating and peak side lobes degrade the performance of some spatial spectrum estimation algorithms and even cause many false alarms in target detection. Many scholars have conducted research to improve this problem. An extended coprime array method was proposed to improve the peak side lobe of the CLAs. The method derived a sufficient condition on the extension factor connected with the location and formation of peak side lobes [6]. Similarly, the approaches based on extended CLAs were developed as semi-coprime arrays [10], three-level and multi-level prime arrays [7, 8, 11, 12]. Additionally, designing an array factor by nulling the grating lobes with known directions can also be a solution for this issue [31]. The above problems and solution ideas are based on incoherent signals, while coherent signals are less considered. For coherent signals, especially co-frequency signals, there are more severe problems. The spatial spectrum output of the co-frequency signals of the CLAs will produce grating lobes equivalent to the spatial spectrum of the targets’ directions, which undoubtedly severely impacts the accurate detection of the targets [14]. High-resolution DOA estimation algorithms like MUSIC [32] and estimating signal parameters via rotational invariance techniques (ESPRIT) [33] will fail because of the source covariance matrix’s rank deficiency when the incident signals are coherent. For coherent target detection, several methods have been proposed, including spatial smoothing (SS) [34] and forward/backward SS (FBSS) [35]. However, the SS technique sacrifices array aperture to attain DOA estimation of coherent signals, while the FBSS approach yields superior estimation precision but the incomplete implementation of signal decoherence. In addition, uniform linear arrays (ULAs) are often given more attention than sparse linear arrays in these methods.

For underwater acoustic signal detection and estimation, the grating lobe effect of the array can be reflected in sparse arrays. For instance, a vector hydrophone linear array performs similarly to a sound pressure array for detecting and estimating underwater acoustic signals, but with a smaller array aperture [36]. Furthermore, the sound pressure and vibration velocity combination exhibits robust anti-isotropic noise capabilities [37–39]. In practical applications, vector hydrophone linear arrays allow for a certain degree of sparsity, but the accompanying grating lobes issues are still expected to be addressed.

In this paper, we develop a DOA estimation algorithm for more than two co-frequency signals using a coprime vector sensor array. The conventional beamformer for the whole coprime array is used as the pre-processor, and the vector cross terms (VCTs) are constructed on two subarrays. The algorithm uses the channel combination of vector hydrophones, and the characteristic data point identification method based on VCTs is designed. In order to improve the accuracy, a queue selection of signals based on inverse beamforming is used to perform filtering of suspicious targets. Finally, the orientation information of real targets can be obtained. The proposed method differs from existing techniques in handling co-frequency signals without spatial smoothing, thereby reducing redundancy. The simulation results demonstrate the effectiveness of the proposed algorithm.

*Notations*: The matrices (vectors) are represented by upper-case (lowercase) bold characters. (⋅)*, (⋅)^*H*^ and (⋅)^*T*^ denote the complex conjugate, conjugate transpose and transpose, respectively. ***I*** stands for the unit matrix. ⊗ represents the Kronecker product.

## Coprime vector sensor array signal model

**Fig 1:**
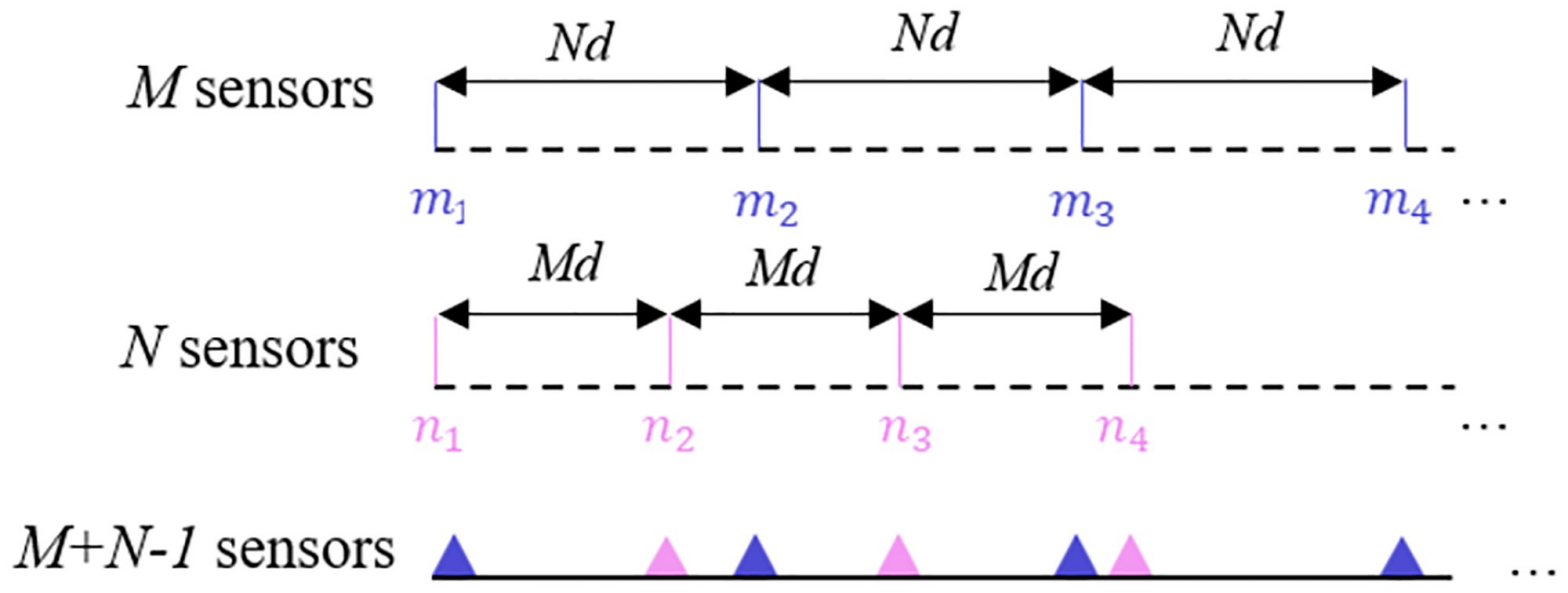
The configuration of a coprime vector sensor array.


An underwater acoustic vector sensor coprime linear array (vCLA) is made up of two sparse uniform subarrays of vector sensors. The first subarray has *M* physical sensors, while the second has *N* physical sensors. It is important to note that *M* and *N* are coprime numbers. In Fig 1, it can be shown that the first subarray, comprising *M* sensors, has an intersensor distance of *Nd*, whereas the second subarray, consisting of *N* sensors, has an intersensor spacing of *Md*. Here, *d* = λ/2 refers to the unit array element spacing, where λ represents the wavelength of the signal impinging on the array. Then the representation of the array configuration is as follows.

$$\begin{array}{c} S=\{Mnd,0\leq n\leq N-1\}\cup \{Nmd,0\leq m\leq M-1\} \end{array}$$ (1)

Supposing that the desired signal comes from direction *θ*_0_, then we can model the received signal as follows.

$$\begin{array}{c} \begin{array}{c} \begin{array}{cc} Y(t) & ={\left[y_{1}\left(t\right),y_{2}\left(t\right),\cdots y_{M+N-1}\left(t\right)\right]}^{T}=a\left(\theta_{0}\right)⊗u\left(\theta_{0}\right)x\left(t\right)+N\left(t\right) \end{array} \end{array} \end{array}$$ (2)

where ***x***(*t*) = [*x*_1_(*t*), *x*_2_(*t*), ⋯ *x*_*M*+*N*−1_(*t*)]^*T*^ refers to the vector of the signal waveform and $N\left(t\right)={\left[n_{1}^{T}\left(t\right),n_{2}^{T}\left(t\right),\cdots n_{M+N-1}^{T}\left(t\right)\right]}^{T}∼CN\left(0,\sigma_{n}^{2}I\right)$ represents the Gaussian noise component with statistical independence and the power of $\sigma_{n}^{2}$. Here ***n***_*i*_(*t*) = [*n*_*p*_(*t*), *n*_*vx*_(*t*), *n*_*vy*_(*t*)]^*T*^, *i* = 1, 2, ⋯ *M* + *N* − 1 is the noise vector, which is composed of the pressure component and the horizontal velocity components, denoted as *n*_*p*_(*t*), *n*_*vx*_(*t*) and *n*_*vy*_(*t*) respectively.

***a***(*θ*) is the steering vector connected with DOA *θ*_0_:

$$\begin{array}{c} a\left(\theta_{0}\right)={\left[1,e^{-j\frac{2\pi }{\lambda }d_{2}\;\text{sin}\left(\theta_{0}\right)},...,e^{-j\frac{2\pi }{\lambda }d_{M+N-1}\text{sin}\left(\theta_{0}\right)}\right]}^{T} \end{array}$$ (3)

**Fig 2:**
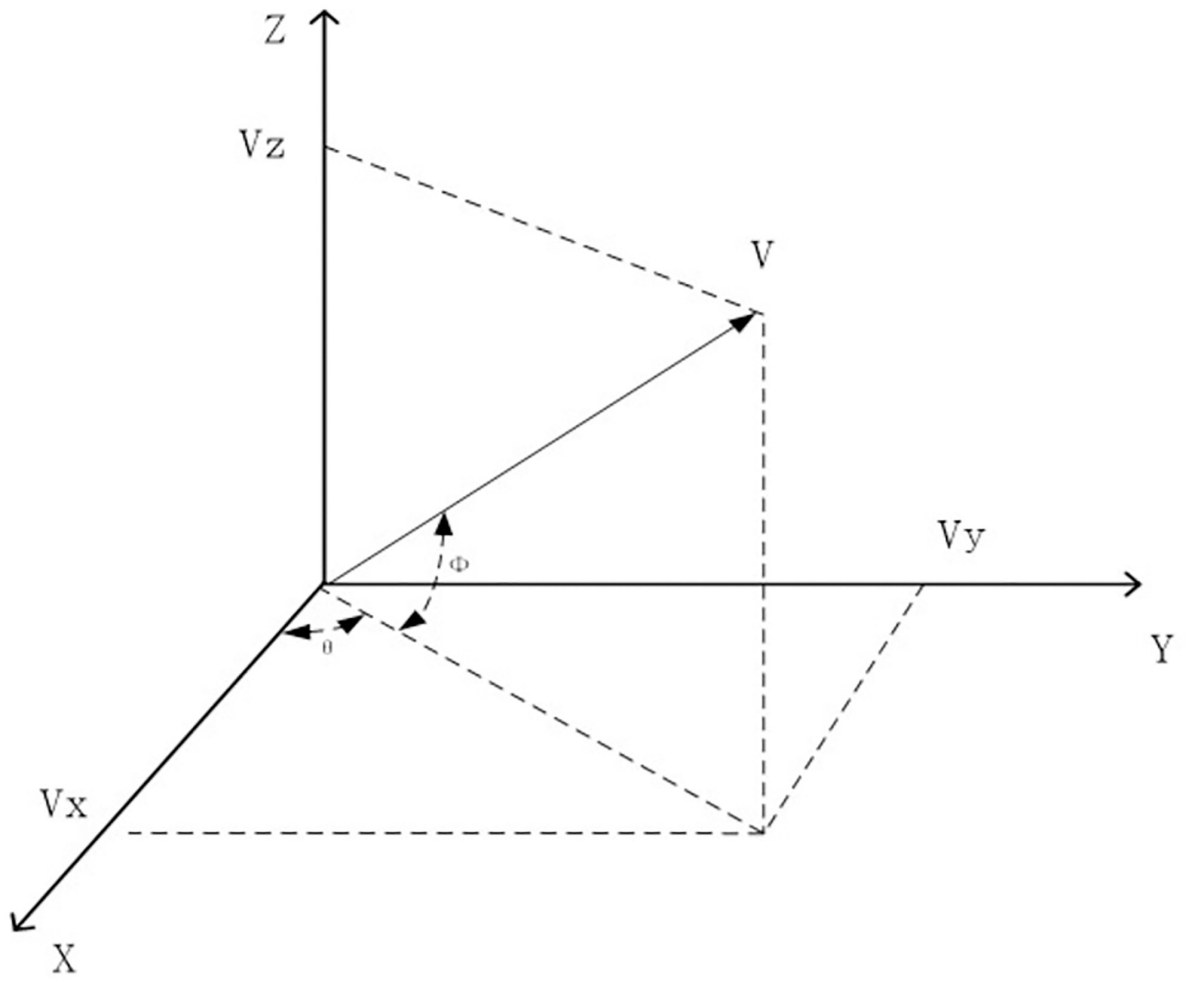
The structure of a vector hydrophone.

The acoustic vector sensor is a device that can simultaneously measure sound pressure and particle velocity at a single point in the sound field, reducing the need for multiple devices and minimizing measurement redundancy. As shown in Fig 2, the relationship between different channels of a vector sensor after rotation can be obtained from the Givens transform as follows:

$$\begin{array}{c} \left\{ \begin{array}{c} \begin{array}{cc} P(t) & =x(t)\\ V_{x}\left(t\right) & =x\left(t\right)\text{cos}\left(\theta_{0}\right)\\ V_{y}\left(t\right) & =x\left(t\right)\text{sin}\left(\theta_{0}\right) \end{array} \end{array}\right. \end{array}$$ (4)

So the 3 × 1 steering vector can be formulated as:

$$\begin{array}{c} u\left(\theta_{0}\right)=\left[1,\text{cos}\left(\theta_{0}\right),\text{sin}\left(\theta_{0}\right)\right] \end{array}$$ (5)

The conventional beamformer (CBF) is given by [40]:

$$\begin{array}{c} B_{vCLA}=\frac{{\left|w_{vCLA}^{H}x_{vCLA}\right|}^{2}}{{\left(M+N-1\right)}^{2}} \end{array}$$ (6)

$$\begin{array}{c} w_{vCLA}=w_{CLA}⊗u\left(\theta \right) \end{array}$$ (7)

$$\begin{array}{c} \omega_{CLA}=e^{-j\frac{2\pi }{\lambda }d_{CLA}sin\left(\theta \right)} \end{array}$$ (8)

where ***w***_*CLA*_ is the weight of beamformer and $d_{CLA}\in S$. ***x***_*vCLA*_ denotes the signal vector of the vCLA. In practical applications, the maximum beam output in the direction of the source can be achieved when there is a single source. Nevertheless, When sources have the same frequency, the significant cross term arises in Eq (6). Consequently, there will be high beam outputs in directions other than the target directions, which could cause false alarms or inaccurate estimations of the DOAs. The cross term can be attributed to the relative positioning of the main lobes and grating lobes in the beam output of two subarrays in a coprime vector sensor array [41]. There is the following relationship along the axis of sin(*θ*).

$$\begin{array}{c} \text{sin}\left(\theta_{N}\right)\pm i_{1}\frac{2}{N}=\text{sin}\left(\theta_{M}\right)\pm i_{2}\frac{2}{M} \end{array}$$ (9)

where *i*_1_ = 0, 1, 2, …, *i*_2_ = 0, 1, 2, …, and *θ*_*N*_ and *θ*_*M*_ are two angles causing false alarms. When the main lobe of one subarray aligns with the grating lobe of the other subarray, or when the grating lobe of one subarray aligns with the grating lobe of the other subarray, the output beams have similar amplitudes to the true sources, leading to ambiguity.

## DOA estimation for multiple coherent sources with same frequency

### The VCTs construction for coprime vector hydrophone array

The proper combination of sound pressure and vibration velocities can form various directivity of a vector hydrophone. A vector hydrophone can effectively reduce noise by combining sound pressure and vibration velocity in the processing of the acoustic vector signal. Therefore, to obtain the desired output, the channels of acoustic vector hydrophones are transformed by rotation and combination methods. The Eq (10) is obtained

$$\begin{array}{c} V_{c}\left(t\right)=V_{x}\left(t\right)\text{cos}\left(\varphi \right)+V_{y}\left(t\right)\text{sin}\left(\varphi \right)=S\left(t\right)cos\left(\theta -\varphi \right) \end{array}$$ (10)

$$\begin{array}{c} \begin{array}{c} V_{s}\left(t\right)=-V_{x}\left(t\right)sin\left(\varphi \right)+V_{y}\left(t\right)cos\left(\varphi \right)=S\left(t\right)sin\left(\theta -\varphi \right) \end{array} \end{array}$$ (11)

Here, *φ* denotes the angle of electron rotation and ***S***(*t*) represents the sound pressure signal. This paper utilizes a combination of sound pressure and vibration velocity:

$$\begin{array}{c} \left[P\left(t\right)+V_{c}\left(t\right)\right]V_{s}\left(t\right)=S^{2}\left(t\right)B_{s}\left(\theta \right) \end{array}$$ (12)

where

$$\begin{array}{c} B_{s}\left(\theta \right)=\left(1+\text{cos}\left(\theta -\varphi \right)\right)\text{sin}\left(\theta -\varphi \right) \end{array}$$ (13)

**Fig 3:**
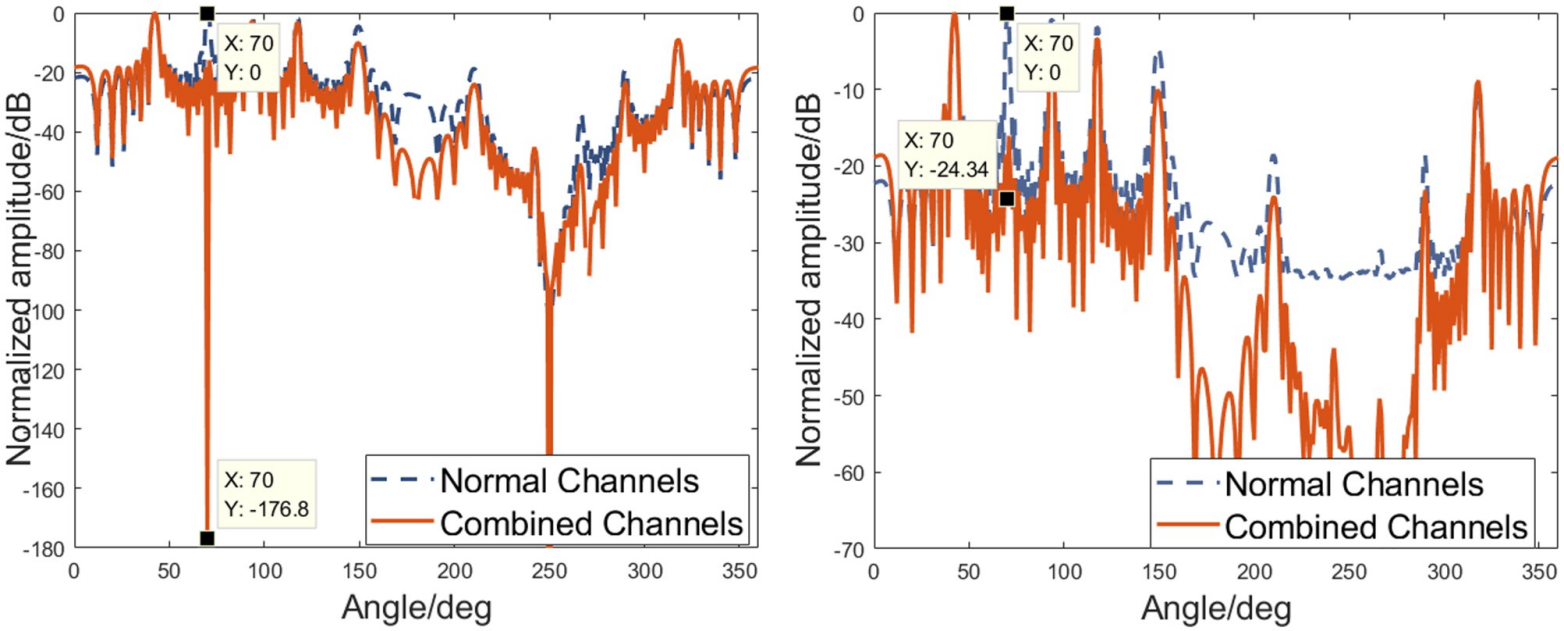
Comparison of processing each channel as normal and combining channels for a vector hydrophone. (a) Spatial spectrum output of a vector sparse array without considering noise. (b) Spatial spectrum output of a vector sparse array with SNR = 20 dB.

It can be seen that ***B***_*s*_(*θ*) = 0 when *θ* = *φ*. The method used for noise reduction by combining sound pressure and vibration velocity processing can be classified as a generalized spatial domain filtering technique. Therefore, selecting a suitable rotation angle *φ* and rotating the acoustic vector hydrophone data can reduce noise and effectively detect weak targets. For a single uniform sparse vector hydrophone array, traditional beamforming based on Eq (13) can generate a spatial spectrum output related to the target direction (take the target from 70° as an example). The output is shown in Fig 3, and “Normal Channels” in the legend represents the sound pressure and velocity channels of vector hydrophones are treated as the same type channels, i.e., the data of each channel is not combined. Without considering noise, the spatial spectrum output based on a vector hydrophone combined channels exhibits a concave point at the source direction, which is shown in Fig 3(a). Unfortunately, the reliability of using concave points to determine target directions may be affected by the presence of noise. What’s worse, for a sparse array, the unreliability can be more severe as the array spacing increases. In this paper, we proposed a DOA estimation method for a coprime vector hydrophone array, which uses vector cross terms (VCTs) inspired by the Product theorem [18, 41]. This method resolves spatial frequency ambiguities by multiplying the response of one subarray with the complex conjugate of the other [1]. Suppose that the vector hydrophones in a subarray consisting of *M* sensors receive the data of the acoustic pressure, x-axial acoustic particle velocity, and y-axial acoustic particle velocity, which can be denoted as ***P***_*M*_(*t*), ***V***_*x*__*M*_(*t*), and ***V***_*y*__*M*_(*t*), respectively. On the other hand, let ***P***_*N*_(*t*), ***V***_*x*__*N*_(*t*), and ***V***_*y*__*N*_(*t*) denote the data of acoustic pressure, x-axial acoustic particle velocity, and y-axial acoustic particle velocity that the vector hydrophones from a subarray with *N* sensors have received. Then we can create the vector cross terms for a coprime vector hydrophone array by using the following construction:

$$\begin{array}{c} \left\{ \begin{array}{c} C_{1}\left(t\right)=\left(P_{M}\left(t\right)+V_{cM}\left(t\right)\right)V_{sN}\left(t\right)\\ C_{2}\left(t\right)=\left(P_{N}\left(t\right)+V_{cN}\left(t\right)\right)V_{sM}\left(t\right) \end{array}\right. \end{array}$$ (14)

where

$$\begin{array}{c} \left\{ \begin{array}{c} V_{cM}\left(t\right)=V_{xM}\left(t\right)\text{cos}\left(\varphi \right)+V_{yM}\left(y\right)\text{sin}\left(\varphi \right)\\ V_{sM}\left(t\right)=-V_{xM}\left(t\right)\text{sin}\left(\varphi \right)+V_{yM}\left(t\right)\text{cos}\left(\varphi \right) \end{array}\right. \end{array}$$ (15)

$$\begin{array}{c} \left\{ \begin{array}{c} V_{cN}\left(t\right)=V_{xN}\left(t\right)\text{cos}\left(\varphi \right)+V_{yN}\left(y\right)\text{sin}\left(\varphi \right)\\ V_{sN}\left(t\right)=-V_{xN}\left(t\right)\text{sin}\left(\varphi \right)+V_{yN}\left(t\right)\text{cos}\left(\varphi \right) \end{array}\right. \end{array}$$ (16)

### Data cross validation based on VCTs

**Fig 4:**
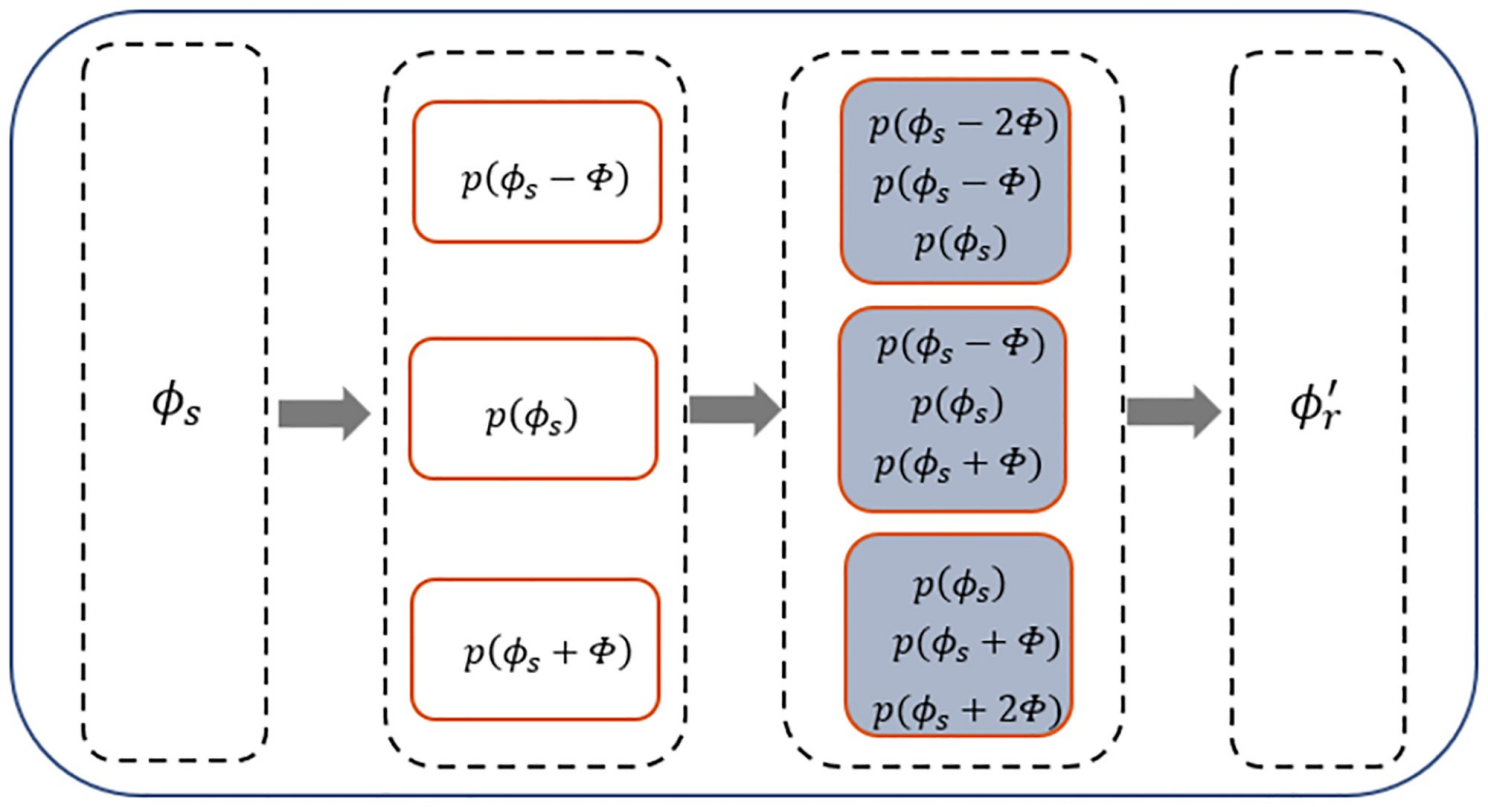
The algorithm diagram for the discriminating process.

The concave point discriminant function we introduce is based on the output of the array spatial spectrum and is designed to identify concave points.

$$\begin{array}{c} p\left(\theta \right)=b_{NM}\left(\theta \right)\cdot {b_{MN}}^{*}\left(\theta \right) \end{array}$$ (17)

where *b*_*NM*_(*θ*) and *b*_*MN*_(*θ*) are the beam outputs calculated by using the covariance of combined data generated by Eq (14). The reliability of concave point judgment can be improved by establishing a relationship between two sparse subarrays of the coprime vector hydrophone array instead of using a single sparse array. The inverse Advance and Retreat method [42] is utilized to detect concave points in the direction of suspected targets. Let Φ be the search step and *ϕ*_*s*_ be the suspected target’s orientation. The discriminating process can be expressed as

$$\begin{array}{c} \;\;D_{p,0}=IF\left(p\left(\phi_{s}\right)-p\left(\phi_{s}-\Phi \right)<0\right)\\ \;\;\;\;\cdot IF(p\left(\phi_{s}\right)-p\left(\phi_{s}+\Phi \right)<0),\\ \;\;D_{p,\Phi }=IF\left(p\left(\phi_{s}+\Phi \right)-p\left(\phi_{s}\right)<0\right)\\ \;\;\;\;\;\;\cdot IF(p\left(\phi_{s}+\Phi \right)-p\left(\phi_{s}+2\Phi \right)<0),\\ \;\;\;\;D_{p,-\Phi }=IF\left(p\left(\phi_{s}-\Phi \right)-p\left(\phi_{s}-2\Phi \right)<0\right)\\ \;\;\;\;\cdot IF(p\left(\phi_{s}-\Phi \right)-p\left(\phi_{s}\right)<0). \end{array}$$ (18)

where ‘*IF*()’ represents an if conditional operation. The algorithm diagram for the discriminating process is shown as Fig 4, where $\phi_{r}^{\prime }$ stands for the orientation result of discriminating. The discriminating process of each orientation point is based on the joint judgment of its surrounding expansion points, and the minimum interval is related to the algorithm resolution.

### Queue Selection (QS) method of multi-frequency coherent targets based on signal energy

For the case of two targets with the same frequency, the method mentioned above can directly obtain the directions of real targets. Nevertheless, when the number of targets exceeds two, the interference between the grating lobes caused by the same frequency signal intensifies, resulting in the inability to obtain the true DOAs directly. In order to reduce this kind of impact, this article uses inverse beamforming cancellation technology to select the estimated direction of arrival one by one in the queue, thereby reducing false alarms caused by interference between co-frequency grating lobes [43]. Let Θ be a set of predicted directions.

$$\begin{array}{c} \Theta =\{\theta_{1},\theta_{2},\theta_{3}\ldots \theta_{S_{1}}\} \end{array}$$ (19)

where *S*_1_ denotes the number of suspected targets obtained based on Eq (18) in the DOA pre-estimation step. For example, assume that an interference target comes in the direction of *θ*_1_. For the subarray with *M* sensors of the coprime vector sensor array, the array element response vector is expressed as:

$$\begin{array}{c} a_{M}\left(\theta_{1}\right)=\left[1,e^{-j\varphi_{M}},e^{-j2\varphi_{M}},\ldots ,e^{-j\left(M-1\right)\varphi_{M}}\right] \end{array}$$ (20)

where *φ*_*M*_ = 2*πf*_0_*Nd*cos(*θ*_1_)/*c*, *f*_0_ is the central frequency of the signal and *c* is the speed of sound. The signal component received by subarray with *M* sensors in the sector where the interference bearing is located can be expressed as:

$$\begin{array}{c} S_{M}\left(t\right)=a_{M}\left(\theta_{1}\right)x\left(t\right) \end{array}$$ (21)

$$\begin{array}{c} V_{x_{M}}\left(t\right)=S_{M}\left(t\right)\text{cos}\left(\theta_{1}\right) \end{array}$$ (22)

$$\begin{array}{c} V_{y_{M}}\left(t\right)=S_{M}\left(t\right)\text{sin}\left(\theta_{1}\right) \end{array}$$ (23)

where ***x***(*t*) is the actual signal. The sector is centered on *θ*_1_ and contains 2Ω + 1 beams. The array weighting matrix corresponding to the *b*-th beam is formulated as:

$$\begin{array}{c} W_{M}\left(\theta_{bM}\right)=\left[1,e^{j\varphi_{bM}},e^{j2\varphi_{bM}},\ldots ,e^{j\left(M-1\right)\varphi_{bM}}\right] \end{array}$$ (24)

where *φ*_*bM*_ = *φ*_*M*_. By inverse beamforming, we obtain:

$$\begin{array}{c} \begin{array}{c} I_{\omega M}=\left[1/M\left(2\Omega +1\right)\right]x\left(t\right)\;\sum_{b=-\Omega }^{\Omega }\;a(\theta_{bM})\sum_{i=1}^{M}e^{\left\{-j\left[\left(i-1\right)\left(\varphi -\varphi_{bM}\right)\right]\right\}} \end{array} \end{array}$$ (25)

When the sector is approximately a small angle sector, *e*^{−*j*[(*i*−1)(*φ*−*φ*_*bM*_)]}^ ≅ 1, then *I*_*ωM*_ = *s*_*m*_. Then we can derive the information for the velocity of the vector array:

$$\begin{array}{c} \begin{array}{c} v_{x_{m}}=\left(1/M\right)W_{M}\left(\theta_{bM}\right)V_{x_{M}}\\ =\left(1/M\right)x\left(t\right)\text{cos}\left(\theta_{1}\right)\sum_{i=1}^{M}e^{\left\{-j\left[\left(i-1\right)\left(\varphi -\varphi_{bM}\right)\right]\right\}} \end{array} \end{array}$$ (26)

$$\begin{array}{c} \begin{array}{c} v_{y_{m}}=\left(1/M\right)W_{M}\left(\theta_{bM}\right)V_{y_{M}}\\ =\left(1/M\right)x\left(t\right)\text{sin}\left(\theta_{1}\right)\sum_{i=1}^{M}e^{\left\{-j\left[\left(i-1\right)\left(\varphi -\varphi_{bM}\right)\right]\right\}} \end{array} \end{array}$$ (27)

Similarly, the operation formulas for the subarray with *N* sensors are as follows:

$$\begin{array}{c} a_{N}\left(\theta_{1}\right)=\left[1,e^{-j\varphi_{N}},e^{-j2\varphi_{N}},\ldots ,e^{-j\left(N-1\right)\varphi_{N}}\right] \end{array}$$ (28)

$$\begin{array}{c} \varphi_{N}=2\pi f_{0}Md\text{cos}\left(\theta_{1}\right)/c \end{array}$$ (29)

$$\begin{array}{c} S_{N}\left(t\right)=a_{N}\left(\theta_{1}\right)x\left(t\right) \end{array}$$ (30)

$$\begin{array}{c} V_{x_{N}}\left(t\right)=S_{N}\left(t\right)\text{cos}\left(\theta_{1}\right) \end{array}$$ (31)

$$\begin{array}{c} V_{y_{N}}\left(t\right)=S_{N}\left(t\right)\text{sin}\left(\theta_{1}\right) \end{array}$$ (32)

$$\begin{array}{c} W_{N}\left(\theta_{bN}\right)=\left[1,e^{j\varphi_{bN}},e^{j2\varphi_{bN}},\ldots ,e^{j\left(N-1\right)\varphi_{bN}}\right] \end{array}$$ (33)

$$\begin{array}{c} \begin{array}{c} I_{\omega N}=\left[1/N\left(2\Omega +1\right)\right]x\left(t\right)\;\sum_{b=-\Omega }^{\Omega }\;a(\theta_{bN})\sum_{i=1}^{N}e^{\left\{-j\left[\left(i-1\right)\left(\varphi -\varphi_{bN}\right)\right]\right\}} \end{array} \end{array}$$ (34)

$$\begin{array}{c} \begin{array}{c} v_{x_{n}}=\left(1/N\right)x\left(t\right)\text{cos}\left(\theta_{1}\right)\sum_{i=1}^{N}e^{\left\{-j\left[\left(i-1\right)\left(\varphi -\varphi_{bN}\right)\right]\right\}} \end{array} \end{array}$$ (35)

$$\begin{array}{c} \begin{array}{c} v_{y_{n}}=\left(1/N\right)x\left(t\right)\text{sin}\left(\theta_{1}\right)\sum_{i=1}^{N}e^{\left\{-j\left[\left(i-1\right)\left(\varphi -\varphi_{bN}\right)\right]\right\}} \end{array} \end{array}$$ (36)

where *φ*_*bN*_ = *φ*_*N*_. The sound pressure and velocity channels are used as general channels to obtain beam data in the interference direction *θ*_1_. Let the signal reception matrix after interference rejection be ***Z***, then the received data of the incident signal after cancellation is:

$$\begin{array}{c} S^{*}=S-Z \end{array}$$ (37)

$$\begin{array}{c} V_{x}^{*}=V_{x}-Z\cdot \text{cos}\left(\theta_{1}\right) \end{array}$$ (38)

$$\begin{array}{c} V_{y}^{*}=V_{y}-Z\cdot \text{sin}\left(\theta_{1}\right) \end{array}$$ (39)

In the process of interference rejection, the grating lobes brought by the interference are also weakened, which plays a role in the discrimination of whether the target is real. Queue interference suppression is performed in the pre-estimated orientation target obtained based on Eq (10) to Eq (18), and then judge whether the pre-estimated directions still exist in the result, so finally, true targets orientations can be obtained. The pseudo-code of this part is presented in Algorithm 1.

**Algorithm 1 Pseudo code of the QS method**.

**Initailize**:

1: Input data: Array signal ***S***, ***V***_*x*_ and ***V***_*y*_, Interference direction *θ*_*i*_

2: Initailize parameters: Signal integral length *T*_*s*_, Angle search range Θ_*s*_

**Main Loop:**

3: **while** *Length*(***S***_*t*_) **do** = *T_s_*

4:  Array element response vector ***a***(*θ_i_*) with Eq (20) and Eq (28)

5:  Inverse beamforming weighting matrix ***W*** with Eq (24) and Eq (33)

6:  Array beam output ***Z*** with Eq (25) to Eq (27) and Eq (34) to Eq (36)

7:  Update Θ_*c*_ with Eq (14) to Eq (18)

8:  Array data after suppression S*, $V_{x}^{*}$ and $V_{y}^{*}$

9:  **for** *i* = 1 : Θ_*s*_ **do**

10:   Beamforming ***B**_i_* with Eq (6)

11:  **end for**

12: **end while**

13: Output $B_{\Theta_{s}}$

**Fig 5:**
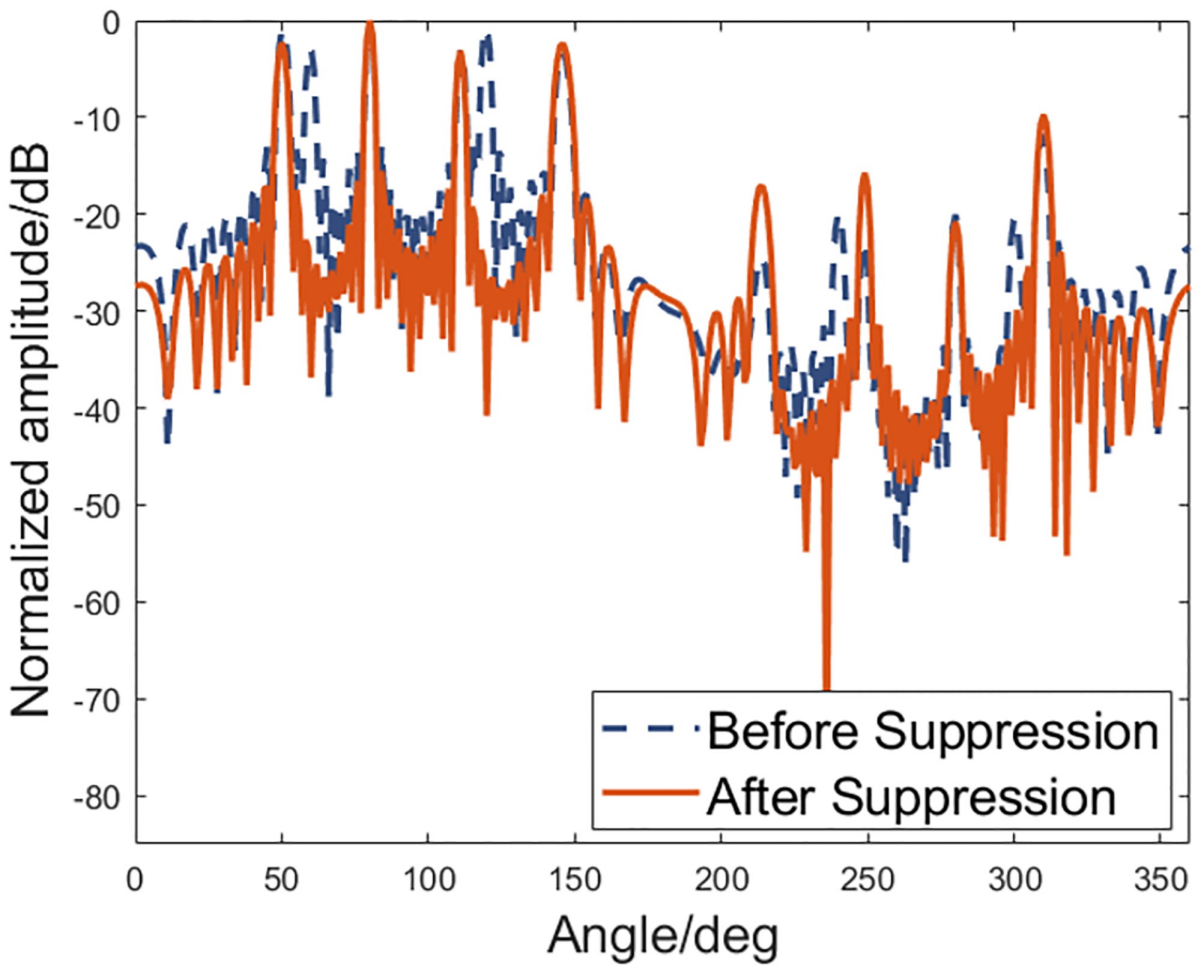
Interference suppression simulation.

For example, as shown in Fig 5, there are two sources and one interference. The directions of two sources are 50° and 80° respectively and the interference comes from 120°. For the sparse vector sensor array, multiple targets and interferences and their grating lobes are in the array spatial spectrum output before interference suppression. After the interference of 120° is suppressed, the grating lobe effect produced by it also disappears, and then the grating lobe suppressing is achieved.

### Major steps and practical application

(1) First, the number of suspected targets and their respective orientations are determined in advance based on Eq (6).

(2) Second, the VCTs and discriminant function to identify concave points are created using Eqs (14) and (17).

(3) The direction of the source can be determined in both scenarios where a single target is present or when two targets are detected with a defined detection threshold. As there is no chance of false alarms in the array’s output in both scenarios, only the actual output is displayed. The target DOA estimation preprocessing can be achieved by Eqs (9) and (18). For more directions of multiple co-frequency signals, the queue selection processing based on Eqs (19) to (39) can be conducted for interference rejection.

(4) Then interference suppression and target orientation verification are performed sequentially to obtain the final target orientation results, which are executed by Eqs (17) to (39). The pseudo code of the overall algorithm is shown in Algorithm 2.

**Algorithm 2 Pseudo code of the major steps for the overall algorithm**.

**Initailize**:

Input data: Array signal ***S***_*t*_

2: Initailize parameters: Detection threshold *D*_*T*_, Signal integral length *T*_*s*_, Target bearing set Θ_1_ and Θ_2_, Concave point set Θ_*c*_, Angle search range Θ_*s*_, the flag for grating lobes exist or not *Flag* = 0

**Main Loop**:

**while** *Length*(***S***_*t*_) = *T*_*s*_ **do**

4:   **for** *j* = 1 : Θ_*s*_ **do**

   Beamforming ***B***_*j*_ with Eq (6)

6:   **end for**

   Output ***B***_Θ_

8:   Update Θ_1_ with *D*_*T*_

   Update *Flag* with Eq (9)

10:  **if** *Flag* = 1 **then**

   Update Θ_*c*_ with Eq (14) to Eq (18)

12:  **if** ((Θ_1_ ∩ Θ_*c*_) ≠ ∅) **then**

    Update Θ_1_

14:   **for** *i* = 1 : Θ_1_ **do**

     Update Θ_2_ with Eq (19) to Eq (39)

16:   **end for**

     **for** *k* = 1 : Θ_2_ **do**

18:     Update Θ_*c*_ with Algorithm 1

     **end for**

20:   **if** ((Θ_2_ ∩ Θ_*c*_) ≠ ∅) **then**

     Update Θ_2_

22:   **end if**

   **else**

24:    *D*_*T*_ Adjustment

   **end if**

26:  **end if**

 **end while**

28: Output Θ_2_

## Simulation and results

**Fig 6:**
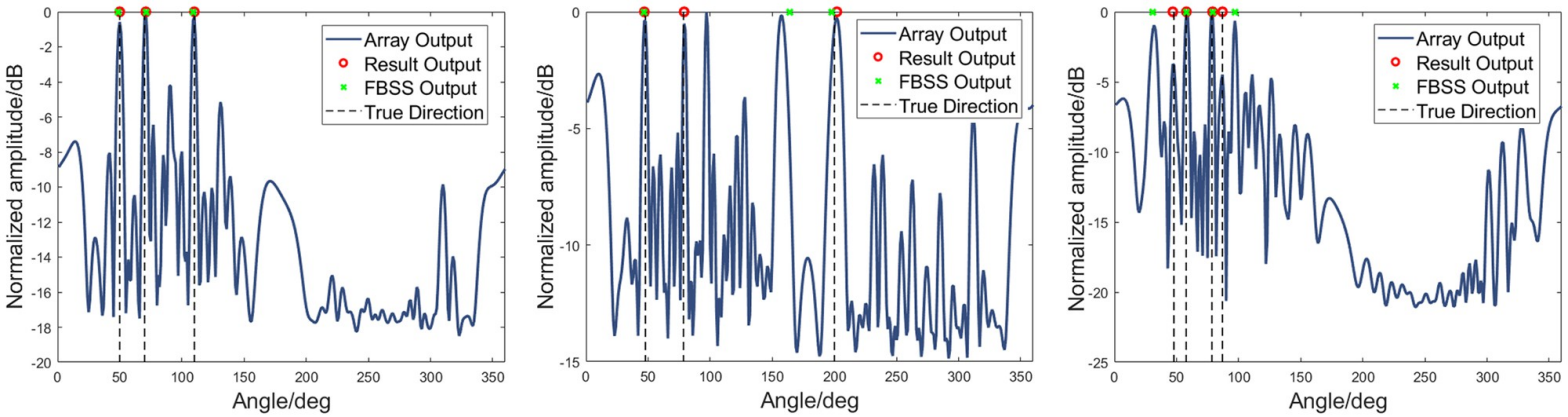
Simulation results. (a) Sources are from 50°, 70° and 110°, SNR = 0 dB. (b) Sources are from 47.9°, 78.5° and 200°, SNR = 0 dB. (c) Sources are from 47.9°, 57.8°, 78.5° and 86.2°, SNR = 5 dB.

Several scenarios were simulated in this part. In the simulation work, a coprime array with *M* = 5 and *N* = 6 sensors for two subarrays, respectively. The number of elements for the whole array is *M* + *N* − 1 = 10. Comparisons between the proposed method and MUSIC based on the FBSS algorithm are included. Firstly, when there are three sources from the directions of 50°, 70°, and 110° respectively, with SNR = 0 dB and *f*=500 Hz. As shown in Fig 6(a), when the three co-frequency targets’ directions do not hold the grating lobes relationship, the array can directly output the accurate orientations without false alarm interferences. The results from both algorithms are consistent with the directions of the actual source. In Fig 6(b), it can be seen that if two of the three targets have a grating lobe relationship and the third target is not in the relationship of the grating lobes with the first two, false alarms can be generated with the same magnitude as the true target orientation spectrums. At this point, the FBSS method produces an incorrect orientation output. By applying the method suggested in this paper, it is possible to identify the correct orientations of the targets. However, there is an error between the target orientations obtained and the actual orientations, as can be seen from Fig 6(b). The algorithm’s error relies on the spatial spectrum output of the whole array. The advantage is that the spatial spectrum output of the whole array ensures that the algorithm has certain robustness. Nevertheless, it can be seen from the figure that when there is an error in the DOA estimation result, the spatial spectrum output of the array already contains the error. In Fig 6(c), two pairs of directions in the four targets satisfy the grating lobe position relationship. It can be seen that the proposed method can extract the actual target orientations with low SNR from the grating lobe interferences. Compared with the traditional decoherence FBSS algorithm, the proposed method does not require decoherence operation. In addition, as shown in Fig 6(c), the proposed method can estimate source orientations more accurately compared with the slightly weaker performance of the FBSS method.

**Fig 7:**
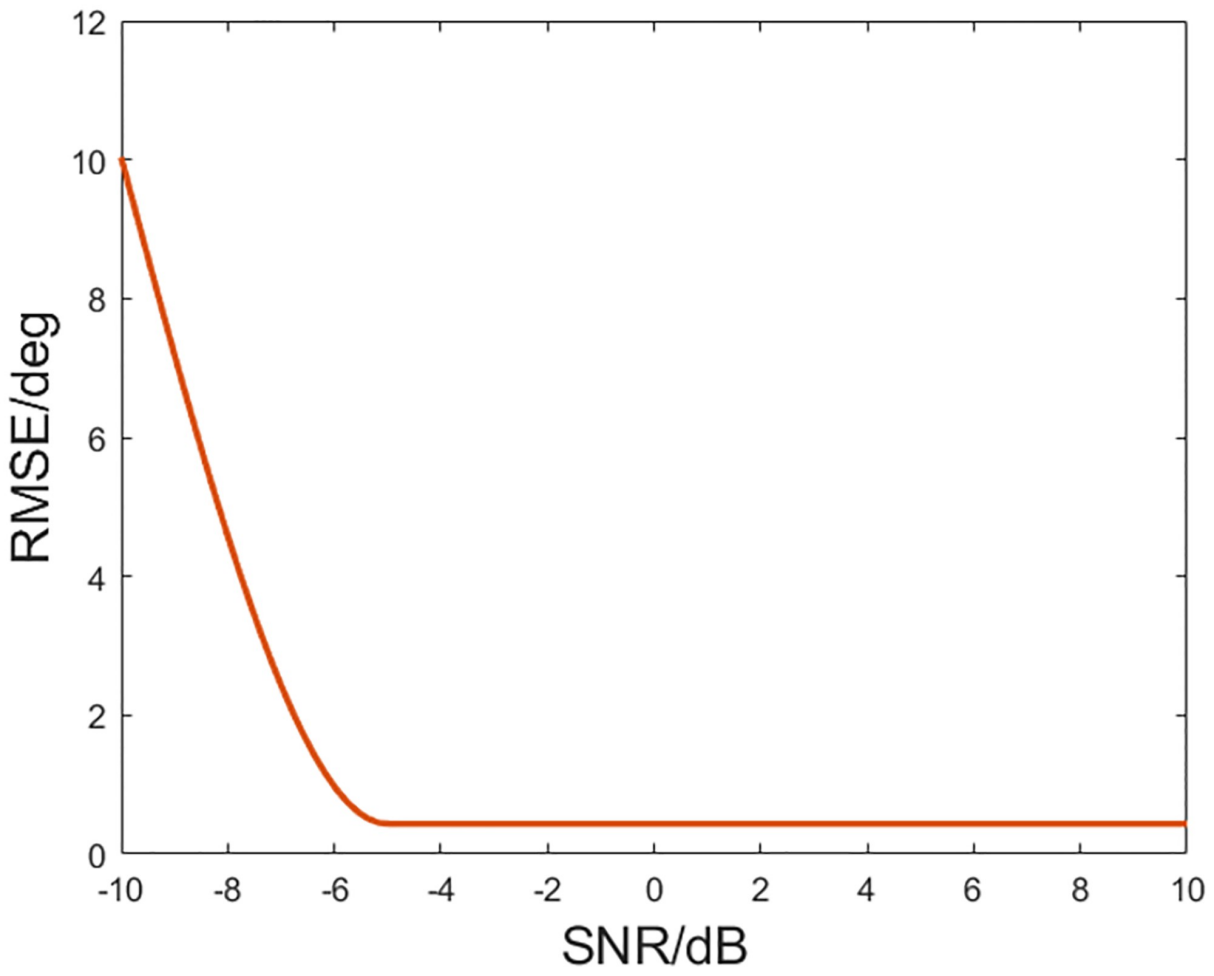
RMSE of DOA estimation.

**Fig 8:**
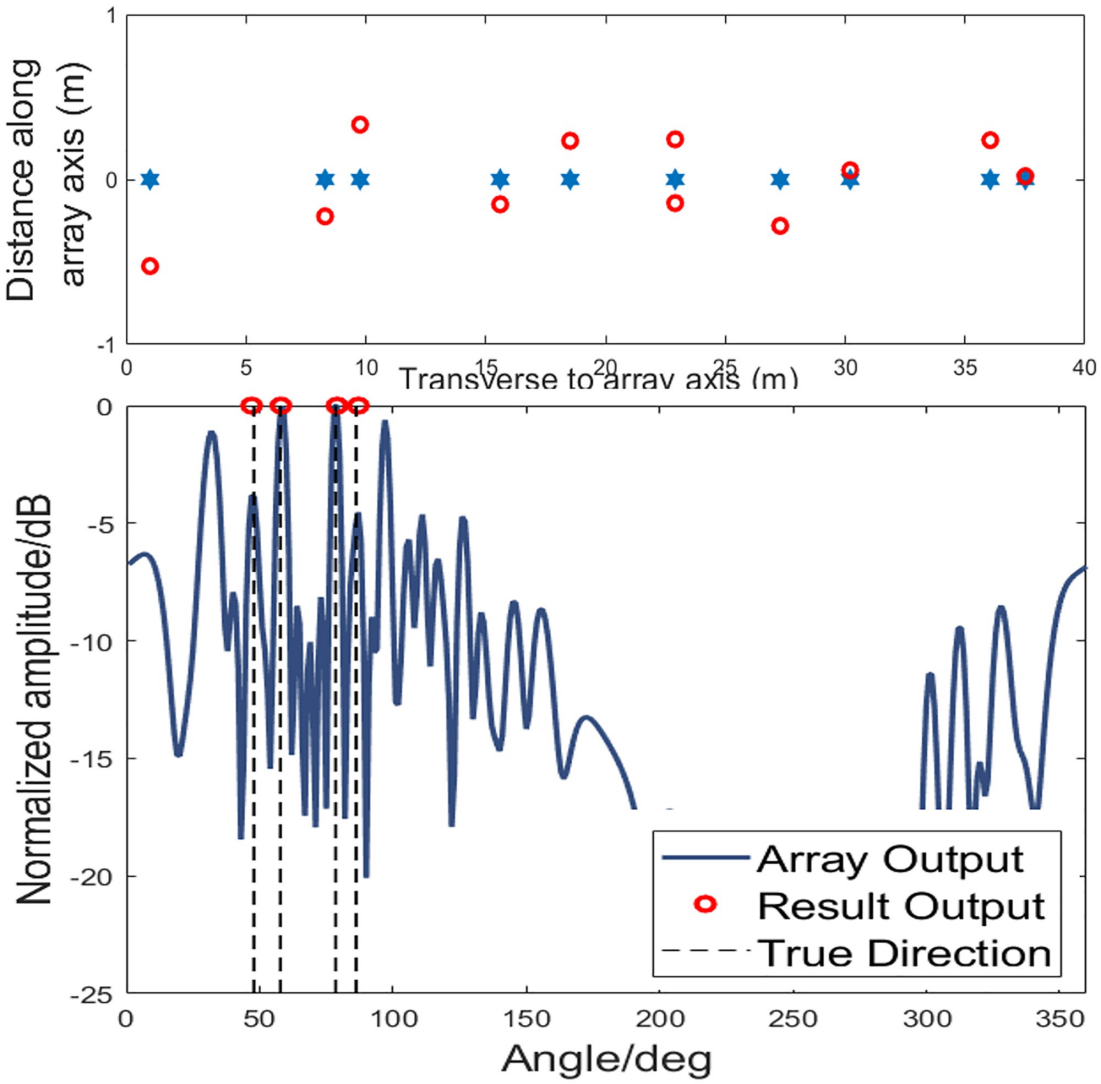
DOA estimation results with array element spacing errors.

The simulation for the existence of the array element position error is carried out to investigate the performance of DOA estimation method in this case. Assume that the array element position error is with a mean of 15% of the spacing between two sensors of the subarray with *N* sensors. The simulation conditions are the same as Fig 6(c). The effect of SNR on the accuracy of proposed method is shown as Fig 7. Each point is averaged based on 100 trials. One can find that starting from the SNR of -5 dB, the algorithm error gradually stabilizes as SNR becomes larger and the error is about 0.5. As shown in Fig 8, one can find that in the presence of array element errors, the algorithm can still obtain the target directions. Because the algorithm is conducted based on the traditional beamforming and thus inherits the robustness of the traditional beamformer.

**Fig 9:**
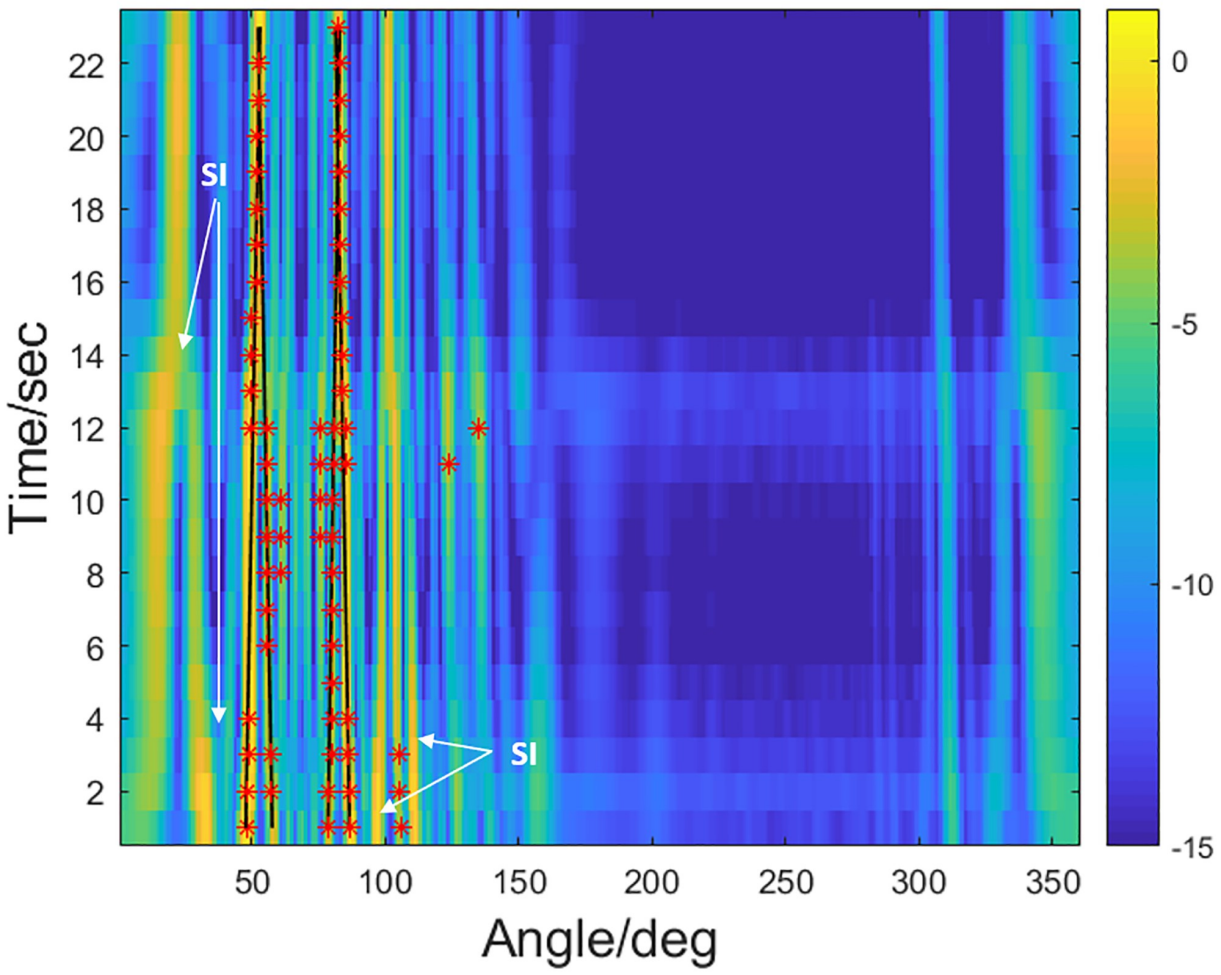
The BTR results with low SNR.

In order to further verify the stability of the algorithm, the simulation of the bearing time record (BTR) with low SNR is carried out. At the same time, the algorithm’s performance for detecting weak targets under interferences is also investigated. In Fig 9, the parts marked with “SI” represent the strong false alarm interference due to the high grating lobes of the array output. The solid black lines represent the true bearing trails of the sources, and the red asterisks exhibit the DOA estimation results with the proposed method. The interferences sources are from the directions of 48° and 86° with SNR = 0 dB, while the true targets come from the directions of 57° and 78° with SNR=-5 dB. The target with the azimuth of 48° is buried in the grating lobe generated by the interference from 78° and the target with 57° is buried in the grating lobe generated by the interference of 86°. Simulation results show that the algorithm can obtain the true orientations of the targets in many interferences. The disadvantage is that a small number of false alarms are inevitably generated due to the strong coherence of the targets.

## Conclusion

In this work, we proposed a method for the direction-of-arrival (DOA) estimation of multiple co-frequency targets using a coprime vector sensor array. The problem that many grating lobes with high amplitudes seriously affect the accurate DOA estimation results was investigated. A conventional beamformer for the coprime vector sensor array as a whole was utilized to preprocess the beam data of the array. After obtaining the set of pre-estimated target orientations, the data cross-validation method based on vector cross terms (VCTs) was then used to transform the detection of the co-frequency targets into a solvable processing without spatial smoothing. We presented the Queue Selection (QS) method based on inverse beamforming cancellation technology to reduce the effect of grating lobe interferences further. The accuracy of directions extraction is enhanced by weakening the grating lobes in a sequential interference suppressing. The simulation results show the reliability and stability of the proposed method. Since the algorithm is based on the traditional beamforming output of the array, the disadvantage is that DOA estimation error will be affected by the array output after beamforming. However, it still inherits the robustness of conventional beamformers.
